# BSDE: barycenter single-cell differential expression for case–control studies

**DOI:** 10.1093/bioinformatics/btac171

**Published:** 2022-03-25

**Authors:** Mengqi Zhang, F Richard Guo

**Affiliations:** Department of Surgery, Perelman Medical School, University of Pennsylvania, Philadelphia, PA 19104, USA; Statistical Laboratory, University of Cambridge, Cambridge CB3 0WB, UK

## Abstract

**Motivation:**

Single-cell sequencing brings about a revolutionarily high resolution for finding differentially expressed genes (DEGs) by disentangling highly heterogeneous cell tissues. Yet, such analysis is so far mostly focused on comparing between different cell types from the same individual. As single-cell sequencing becomes cheaper and easier to use, an increasing number of datasets from case–control studies are becoming available, which call for new methods for identifying differential expressions between case and control individuals.

**Results:**

To bridge this gap, we propose barycenter single-cell differential expression (BSDE), a nonparametric method for finding DEGs for case–control studies. Through the use of optimal transportation for aggregating distributions and computing their distances, our method overcomes the restrictive parametric assumptions imposed by standard mixed-effect-modeling approaches. Through simulations, we show that BSDE can accurately detect a variety of differential expressions while maintaining the type-I error at a prescribed level. Further, 1345 and 1568 cell type-specific DEGs are identified by BSDE from datasets on pulmonary fibrosis and multiple sclerosis, among which the top findings are supported by previous results from the literature.

**Availability and implementation:**

R package BSDE is freely available from doi.org/10.5281/zenodo.6332254. For real data analysis with the R package, see doi.org/10.5281/zenodo.6332566. These can also be accessed thorough GitHub at github.com/mqzhanglab/BSDE and github.com/mqzhanglab/BSDE_pipeline. The two single-cell sequencing datasets can be download with UCSC cell browser from cells.ucsc.edu/?ds=ms and cells.ucsc.edu/?ds=lung-pf-control.

**Supplementary information:**

Supplementary data are available at *Bioinformatics* online.

## 1 Introduction

Single-cell RNA sequencing (scRNAseq) aims at profiling the gene expression in every cell of a given sample, by sequencing their genomes, transcriptomes or proteomes. As such, it overcomes the limitation of the bulk analysis and enables researchers to inspect the spatial–temporal details of a biological procedure with high resolutions. With this technology, the type and the life-cycle status of each cell can be observed and traced. Due to the complex nature of biological procedures, to better understand the mechanisms behind, single-cell sequencing is instrumental in detecting cell heterogeneity, finding rare cell types, selecting specialized biomarkers and characterizing rare molecular features at the cellular level (Giladi and Amit, 2018).

One common strategy for understanding the intrinsic and extrinsic biological processes in scRNAseq is to detect the differentially expressed (DE) genes. Through such analyses, the signal from a certain cell type can be isolated and examined. Yet, there are some challenges. For example, scRNAseq data are highly heterogeneous and usually come with a large number of zero counts, which complicates statistical modeling and analysis.

**Fig. 1.**
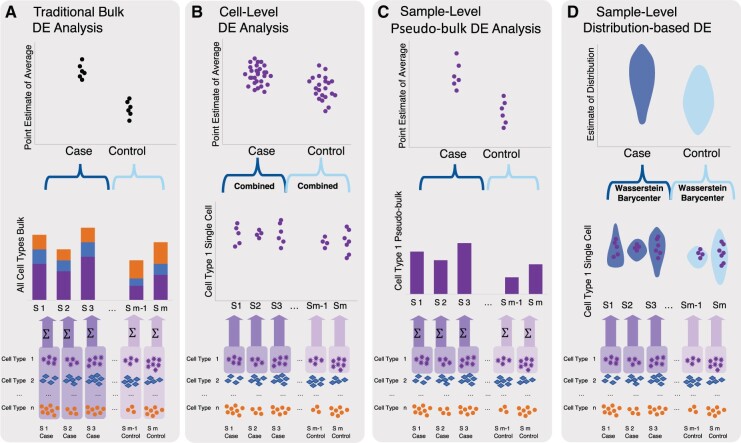
Methods for differential expression analysis can be divided into four categories: (**A**) Traditional bulk DE analysis methods (e.g. DESeq2 and edgeR) compare cases with controls from bulks of cells. The expressions are represented as per gene, per individual; cell type is ignored. (**B**) Cell-level DE analysis methods simply combine each cell as a sample and compare the expression levels between case cells and control cells. Many purpose-built methods belong to this category, including SCDE, MAST, EMDomics, D3E, Monocle, SINCERA, DEsingle and SigEMD. (**C**) Sample-level pseudo-bulk analysis methods (e.g. aggregateBioVar and MUSCAT) combines (A) and (B) by executing in two steps. First, expressions per gene, per individual of a given cell cluster are summarized, which essentially converts data into a bulk format. Then methods from (A) are applied to the summarized data. (**D**) Sample-level distribution-based DE analysis (our method BSDE) aggregates the expressions of a given cell type across cells and individuals into a distribution. The aggregated case distribution and control distribution are compared for identifying differential expressions

In a bulk RNAseq analysis, the overall expression level is point estimated by a count (Category A of Fig. 1). Alternatively, the single-cell data, which contain more information, are represented as an empirical distribution over counts, where each cell contributes a count (Categories B–D of Fig. 1). Naturally, there could be two levels of comparison: cell level and individual level.

First, two cell types from an individual can be compared through their distributions. There are already quite a few methods available from the literature for this purpose, including off-the-shelf statistical tests, such as Mann–Whitney *U*-test, as well as purpose-built parametric and non-parameteric methods, such as SCDE (Kharchenko *et al.*, 2014), MAST (Finak *et al.*, 2015), scDD (Korthauer *et al.*, 2016), EMDomics (Nabavi *et al.*, 2016), D3E (Delmans and Hemberg, 2016), Monocle (Trapnell *et al.*, 2014; Qiu *et al.*, 2017), SINCERA (Guo *et al.*, 2015), edgeR (Robinson *et al.*, 2010), DESeq2 (Love *et al.*, 2014), DEsingle (Miao *et al.*, 2018) and SigEMD (Wang and Nabavi, 2018), to name a few. In terms of the taxonomy given in Figure 1, most of them fall into Category B, except for those originally designed for bulk analysis (edgeR and DESeq2) that belong to Category A.

Further, DE analysis can be performed between two groups of individuals, which is the focus of our paper. In contrast to bulk analysis, existing methods for this type of comparison are scarce; see Zhang *et al.* (2022) for a recent proposal. Some earlier proposals, such as MUSCAT (Crowell *et al.*, 2020) and aggregateBioVar (Thurman *et al.*, 2021), are based on summarizing counts from certain single-cell sequences into a ‘pseudo-bulk’ RNAseq (Category C of Fig. 1). Then, methods from Category A are immediately applicable to these summarized counts; however, as we will see from simulations, summarization discards distributional information and hence cannot detect nuanced differential expressions. At the moment, arguably the most common approach towards such a comparison is based on mixed-effect models. For example, the hurdle model (e.g. Finak *et al.*, 2015), which specifies a logistic regression model for the expression rate and a linear model for the logarithmic non-zero expression, can be fitted with a mixed effect. Fixed effects are fitted on the case/control indicator (along with other covariates), and random effects are fitted by introducing individual-level random intercepts; see Velmeshev *et al.* (2019, Supp. Mat.) for such an analysis on autism data. Yet, performing valid statistical inference (e.g. testing DE at a prescribed significance level) for the fitted mixed-effect model can be challenging. As we will show in Section 3.2, due to the presence of random effects, standard likelihood ratio tests are typically inapplicable to these settings. Further, the suitability of the hurdle model is limited by its parametric assumptions, which may not hold in real data.

To adapt DE analysis to conventional case–control studies, we propose barycenter single-cell differential expression (BSDE), which performs comparison in two stages. The first stage is to aggregate individual-level distributions into a case group distribution and a control group distribution by finding the corresponding Wasserstein barycenters. The second stage is to compare the two group-level distributions in terms of their Wasserstein distance. The Wasserstein barycenter and distance are defined nonparametrically in terms of optimal transportation of probability measures, which does not rely on restrictive parametric assumptions. The type-I error can be readily controlled with a permutation *P*-value or its Monte Carlo approximation.

It is worth mentioning that BSDE, by design, differentiates itself from other methods by comparing distributions instead of simple summary statistics of distributions (e.g. mean). To illustrate the differences, we propose a taxonomy of current methods as shown in Figure 1. We also note that recently Wasserstein distance (or the earth mover’s distance) has been introduced for differential expression analysis; see, e.g., Nabavi and Beck (2015), Nabavi *et al.* (2016) and Wang and Nabavi (2017, 2018), which employ the distance as a test statistic for comparing distributions. However, we argue that our method goes one step further in utilizing the tools from optimal transportation—the case and control distributions themselves are *aggregated* from the individual level as their respective Wasserstein barycenters.

## 2 Materials and methods

We propose BSDE, a nonparametric procedure based on optimal transportation of probability distributions. For two distributions, their Wasserstein distance (also known as the earth mover’s distance) is defined as the minimal cost (in terms of some cost/loss function) of ‘transporting’ the mass of one distribution to the other. Throughout, we will focus on the Wasserstein distance *W*_2_ between distributions *P* and *Q*, defined as

$$W_{2}(P,Q)={\left\{\underset{\gamma \in \Pi (P,Q)}{\min}c(x,y)d\gamma \right\}}^{1/2},$$

where the loss function $c(x,y)=||x-y|{|}^{2}$ is the square Euclidean distance. The set Π(P,Q) refers to the set of couplings between probability distributions *P* and *Q*, i.e., the set of bivariate distributions with *P* and *Q* as margins. Although other loss functions *c*(*x*, *y*) can be considered in principle as well, the square loss is a safe default choice (similar to being the default for regression problems) and its properties are well studied.

Defined as such, it can be argued that the Wasserstein distance is more informative than other definitions of distance or divergence (e.g. total variation, Kullback–Leibler) between distributions as it takes account of the *metric information* captured by the loss function, which in our context, translates to the difference in expression levels.

**Fig. 2.**
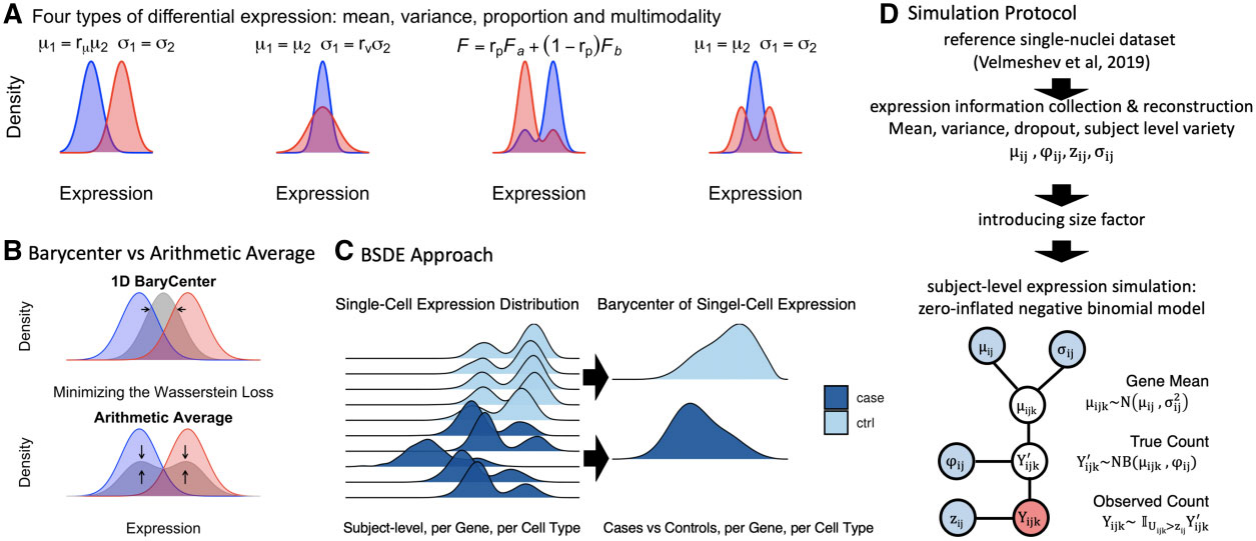
Description of the method and the simulation protocol. (**A**) Four types of differential expression in single-cell sequencing that are considered in simulations. (**B**) Wasserstein barycenter versus arithmetic averaging. Barycenter minimizes the total cost of ‘moving distributions to the averaged distribution. (**C**) BSDE aggregates case/control distributions by finding their respective Wasserstein barycenters. Then, the Wasserstein distance of the two group-level distributions is compared to permutation counterparts for testing significance. (**D**) The simulation protocol roughly follows that of Lun and Marioni (2017)

This further induces a notion of average for a set of distributions. Let $P_{1},\ldots ,P_{n}$ be a collection of distributions on metric space X. Their Wasserstein barycenter is defined as the minimizer to

$$\underset{\mu }{\min}\sum_{i=1}^{n}W_{2}^{2}(\mu ,P_{i}),\;\mu \in P(X),$$

where P(X) is the set of probability measures on X. Note that the square loss is strongly convex and the Wasserstein barycenter is uniquely defined (Agueh and Carlier, 2011, Proposition 3.5). Compared to the arithmetic average of distributions, the Wasserstein barycenter, by additionally using the metric information, better aligns with our intuition of an averaged distribution; see Figure 2B for an illustration.

Wasserstein distance and barycenter enjoy many appealing properties (Villani, 2009) and find applications in various domains, including image processing (Gramfort *et al.*, 2015), computer graphics (Rabin *et al.*, 2011), and very recently, computationally biology (Schiebinger *et al.*, 2019).

As depicted in Figure 2C, BSDE proceeds in two stages.

1. **Distribution aggregation**. Suppose there are *l* cases and *n* controls. Let $P_{1},\ldots ,P_{l}$ be the empirical histograms of case data and $Q_{1},\ldots ,Q_{n}$ be the empirical histograms of control data. The histograms are typically built on the count data under the commonly used x↦ log(x+1) transform to reduce skewness. Further, to ease computation, the histograms are built with a common set of breakpoints. Let $\hat{P}$ and $\hat{Q}$ be the respective Wasserstein barycenters:
  $$\begin{array}{c} \hat{P}={\text{argmin}}_{\mu }\sum_{i=1}^{l}W_{2}^{2}(\mu ,P_{i}),\\ \hat{Q}={\text{argmin}}_{\nu }\sum_{j=1}^{n}W_{2}^{2}(\nu ,Q_{j}), \end{array}$$
  where *μ* and *ν* are minimized over 1D probability distributions, which, without loss of generality, can also be restricted to the set of histograms with the given breakpoints. In practice, to speed up computation, entropy-regularized versions of $\hat{P}$ and $\hat{Q}$ are computed with the Sinkhorn–Knopp matrix scaling algorithm (Benamou *et al.*, 2015), for which we use the implementation provided by Python package POT (Flamary *et al.*, 2021).
2. **Distribution comparison**. Our test statistic is simply taken to be the Wasserstein distance between the two aggregated histograms:
  $$\hat{\lambda }=W_{2}(\hat{P},\hat{Q}),$$
  which is computed with the fast Greenkhorn algorithm (Altschuler *et al.*, 2017).

We reject the null-hypothesis of no differential expression between case and control for larger values of $\hat{\lambda }$. We use permutation to control the type-I error. Under the null hypothesis, the case and control labels can be permuted without changing the distribution of statistic $\hat{\lambda }$. In fact, under the null, the statistics computed under permutations are exchangeable. The *P*-value can be approximated by taking a large number of random permutations. Let $\lambda^{\left(1\right)},\ldots ,\lambda^{\left(N\right)}$ be the statistic computed from *N* (e.g. *N *=* *1000) random permutations. The *P*-value is approximated as

$$p=\frac{1+\sum_{i=1}^{N}I\{{\hat{\lambda }}^{\left(i\right)}\geq \hat{\lambda }\}}{1+N}.$$

The method is implemented in R package BSDE, available from https://github.com/mqzhanglab/BSDE.

## 3 Results

In what follows, we compare BSDE with a number of competing methods on simulated and real datasets.

### 3.1 Methods for comparison

In view of the taxonomy given by Figure 1, we consider the following methods for comparison.

1. MAST (glm, Finak *et al.*, 2015), a mixed-effect model for the cell-level DE analysis. We fit the model in R with b0=MAST::zlm(formula =∼diagnosis,sca = sca, method =‘glmer’, ebayes = FALSE,parallel = TRUE) and conduct inference with MAST::lrTest(b0,‘diagnosis’), where diagnosis represents the case/control label.
2. MAST (mixed effect, Finak *et al.*, 2015), a slight variation of the previous method. The model is fitted with b1=MAST::zlm(formula =∼diagnosis + (1—ind),sca = sca, method =‘glmer’, ebayes = FALSE,parallel = TRUE) and the test is called with MAST::lrTest(b1,‘diagnosis’). Here, ind represents the individual label and diagnosis represents the case/control label. Though not recommended by the software manual, this type of analysis is seen from the literature (e.g. Schirmer *et al.*, 2019; Velmeshev *et al.*, 2019).
3. DESeq2 (Love *et al.*, 2014), a state-of-the-art method for bulk RNAseq analysis. We treat the sum of raw counts from all cells of each individual as a ‘bulk count’.
4. aggregateBioVar (Thurman *et al.*, 2021) sums up counts from certain cell types to form the ‘pseudo-bulk’ counts and then applies DESeq.
5. MUSCAT (Crowell *et al.*, 2020) is also a method based on pseudo-bulk counts. We perform DE analysis using function pbDS provided in their R package.
6. Mann–Whitney *U*-test on the cell level. Expression from each cell is treated as an independent observation.
7. Mann–Whitney *U*-test on the subject (pseudo-bulk) level.

### 3.2 Simulations

#### 3.2.1 Simulation protocol

The simulation protocol is illustrated in Figure 2D. We generate 3000 genes from a particular type of cell of *n* case subjects and *n* control subjects (*n *=* *5, 10, 20), with each subject having *m* cells (m=20,50,100,200,400). We simulate the basic parameters (mean, dispersion and dropout) by drawing from a distribution fitted with a reference dataset, the scRNAseq data on single-nuclei genomics of autism (Velmeshev *et al.*, 2019). Given a set of basic parameters, expression levels are simulated from a zero-inflated negative binomial model.

More concretely, consider simulating the expression levels of gene *i*. For each individual *j*, we estimate parameters ${\hat{\mu }}_{ij}$ (mean), ${\hat{\varphi }}_{ij}$ (dispersion), ${\hat{z}}_{ij}$ (dropout rate) and ${\hat{\sigma }}_{ij}$ (cell level variability) from the reference dataset (on the logarithmic scale). To capture the variability of these parameters across individuals, we fit a four-variate Gaussian distribution. Then, the expression level of gene *i* on the *k*th cell of individual *j*, denoted by *Y_ijk_* in Figure 2D, is simulated from zero-inflated negative binomial model $\text{ZINB}(\mu_{\mathrm{ijk}},\varphi_{ij},z_{ij})$, where $\left(\mu_{ij},\varphi_{ij},z_{ij},\sigma_{ij}\right)$ is drawn from the four-variate Gaussian (on the logarithmic scale) and further $\mu_{\mathrm{ijk}}∼N(\mu_{ij},\sigma_{ij}^{2})$ for each cell *k*.

#### 3.2.2 Types of differential expression

We introduce four types of DE in our simulated data, where the size of each type is controlled by a factor *r*; see Figure 2A.

1. Mean DE: The size factor is varied from $r_{\mu }=1.1,1.2,1.5,2,4$. Parameters $\left(\mu_{*},\varphi_{*},z_{*}\right)$ are specified relative to (μ,φ,z) as
  $$\mu_{*}=\frac{\mu }{r_{\mu }},\;\varphi_{*}=\frac{\varphi \mu }{\mu +(1-r_{\mu })\varphi },\;z_{*}=z$$
  such that case and control variances are the same.
2. Variance DE: The size factor is varied from $r_{v}=1.1,1.2,1.5,2,4$. Parameters $\left(\mu_{*},\varphi_{*},z_{*}\right)$ are specified relative to (μ,φ,z) as
  $$\mu_{*}=\mu ,\;\varphi_{*}=\frac{\varphi \mu }{r_{v}\mu +(r_{v}-1)\varphi +(r_{v}-1)z\varphi \mu }$$
  and $z_{*}=z$ such that the mean remains the same.

3.Proportion DE: The size factor is varied from $r_{p}=0.6,0.7,0.8,0.9$. Counts are simulated from either mixture $r_{p}\text{ZINB}(\mu_{1},\varphi ,z)+(1-r_{p})\text{ZINB}(\mu_{2},\varphi ,z)$ or the component-swapped $\left(1-r_{p})\text{ZINB}(\mu_{1},\varphi ,z)+r_{p}\text{ZINB}(\mu_{2},\varphi ,z\right)$.
4.Multimodality DE: The size factor is varied from $r_{m}=0.1,0.2,0.3,0.4$. Counts are simulated from either a two-component mixture 12$\text{ZINB}(\mu_{1},\varphi ,z)\;+\;$12$\text{ZINB}(\mu_{2},\varphi ,z)$, or its single-component counterpart $\text{ZINB}(\mu_{*},\varphi_{*},z_{*})$. The parameters are related by
  $$\mu_{1}=\frac{\mu_{2}(1+r_{m})}{1-r_{m}}$$
  and
  $$\mu_{*}=\frac{\mu_{2}}{1-r_{m}},\;\varphi_{*}=\frac{\varphi }{1+r_{m}^{2}+r_{m}^{2}\varphi^{2}},\;z_{*}=z$$
  such that the mean and the variance are unchanged.

#### 3.2.3 Results

We compare BSDE with competing methods in terms of (i) the type-I error under the null hypothesis of no differential expression between case and control and (ii) the detection power under the four types of differential expression considered. The significance level is chosen to be 0.05. The power is defined as the proportion of *P*-values no more than 0.05. For more details on the simulation, the reader is referred to https://github.com/mqzhanglab/BSDE\_pipeline.

**Fig. 3:**
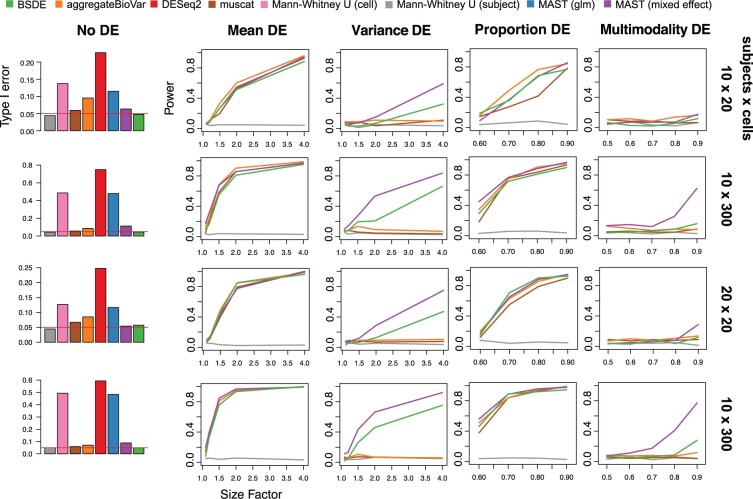
Selected simulation results for comparing BSDE and competing methods. Columns: type-I error (nominal level 0.05 is marked by the red line) under the null hypothesis (no differential expression) and power under four types of differential expression considered in Section 3.2.2. Rows: different settings for the number of subjects and the number of cells; results from more settings are available in the Supplementary Material

The results are presented in Figure 3, where rows correspond to different settings of *n *×* m*; additional settings can be found in the Supplement. From the left panel, we can see that BSDE and the subject-level Mann–Whitney *U*-test are the only two methods that control the type-I error at the nominal level. In particular, as mentioned in Section 1, the uncorrected likelihood ratio-based inference for MAST fails to control the type-I error due to the presence of random effects; the model-based inference for DESeq2 is also found to exceed the nominal level possibly due to misspecification of the parametric model.

In terms of the detection power, strictly speaking, it is only fair to compare methods that maintain the type-I error guarantee. The subject-level Mann–Whitney test hardly has any power. In contrast, BSDE seems to be able to detect differential expression with excellent power in all cases. In particular, we find that the differential expression in variance seems challenging to most of the methods—those based on bulk or summary ‘pseudo-bulk’ counts are unable to detect these signals. The only other method that seems powerful is MAST (mixed effect), which unfortunately does not tightly control the type-I error.

### 3.3 Analysis of pulmonary fibrosis and multiple sclerosis

To demonstrate the use of BSDE on real data, we take two public, case-control study datasets from single-nucleus sequencing: the pulmonary fibrosis (PF) dataset (Habermann *et al.*, 2020, GSE135893) and the multiple sclerosis (MS) dataset (Schirmer *et al.*, 2019, PRJNA544731). The PF dataset contains 20 cases and 10 controls; the MS dataset contains 12 cases and 9 controls. The data were collected with 10× Genomics Single-Cell 3′ system and were preprocessed with software CellRanger. The cell types and meta-information were annotated. We imported data from the matrices with Unique Molecular Identifier counts, with additional normalization and log transformation.

#### 3.3.1 Results on PF

**Fig. 4.**
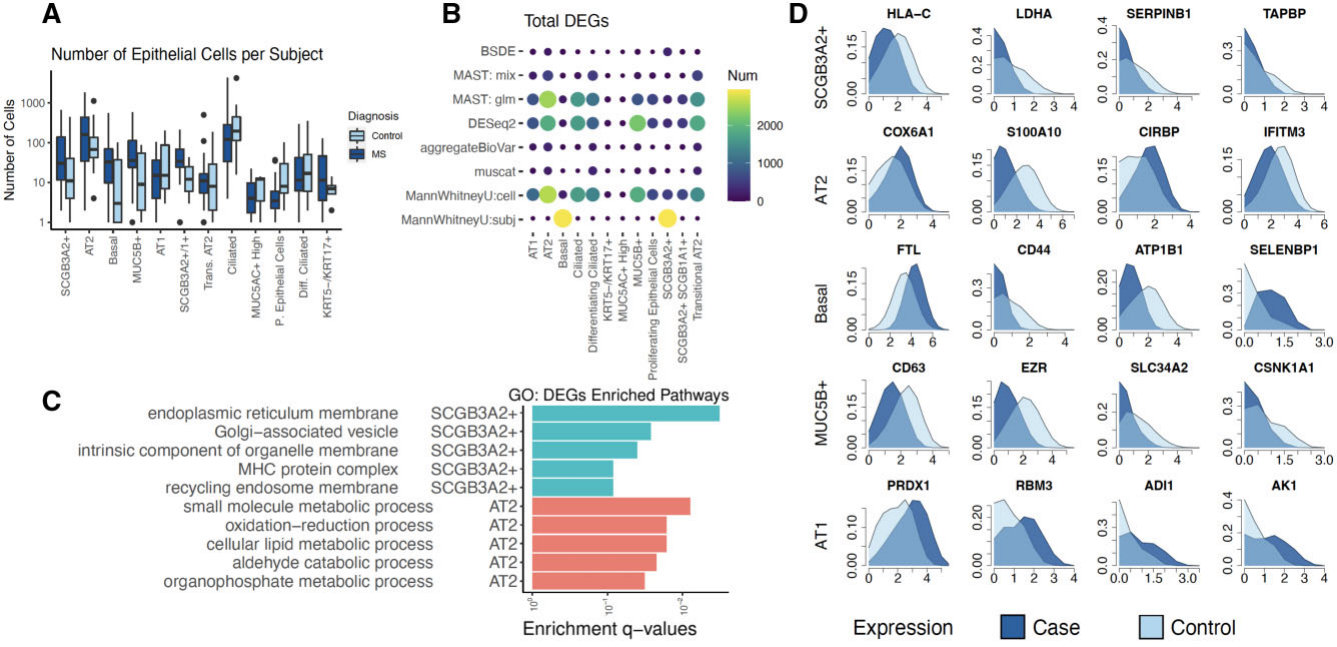
Analysis of the pulmonary fibrosis (PF) dataset. (**A**) Subject-level distributions of the number of cells from case and control samples. (**B**) Number of differentially expressed genes identified by BSDE and competing methods. (**C**) Significant pathways of Gene Ontology (GO) enrichment analysis within the top six epithelial cell types. (**D**) Case and control Wasserstein barycenter distributions for the top four differentially expressed genes (log-transformed expression)

We summarize the results in Figure 4. Figure 4A shows the subject-level distributions of the number of cells from case and control samples. There is no significant difference (*P*-value = 0.16, two-sided Bonferroni-corrected *t*-test) in the numbers of cells between case and control. Figure 4B displays the differentially expressed genes (DEGs) in epithelial cells detected by BSDE. Most signals are found in epithelial cell types SCG3A2+, AT2, Basal, MUC5B+ and AT1. Among these cell types, we perform Gene Ontology (GO) enrichment analysis; the significant pathways are reported in Figure 4C. Further, within each of the aforementioned cell types, in Figure 4D, we contrast the Wasserstein barycenter distributions between case and control for the top four DEGs. Only those genes with a median expression level above four are ranked.

##### Support from the literature

In light of these findings, we review previous results from the literature on the identified cell types and DEGs related to PF; see Table 1 and Table S1 (Supplementary Materials). In particular, DEG-enriched cell types SCGB3A2 (Cai and Kimura, 2015; Zuo *et al.*, 2020), AT2 (Parimon *et al.*, 2020; Wu *et al.*, 2020), MUC5B (Peljto *et al.*, 2013; Seibold *et al.*, 2011; Zuo *et al.*, 2020) and Basal (Carraro *et al.*, 2020; Richeldi *et al.*, 2017) have been previously reported.

**Table 1.** Previous reports of DEG-enriched cell types in PF that are identified by BSDE

Cell type	Previous reports	References
AT2	Tension-activated TGF-beta signaling in AT2 cells	Wu *et al.* (2020)
AT2	Apoptotic death of AT2 cells in PF patients, introduced by ER stress and mitochondrial dysfunction.	Parimon *et al.* (2020)
Basal	Abnormal dysfunction of basal cells in PF.	Richeldi *et al.* (2017)
Basal	Basal cells are dynamically regulated in PF.	Carraro *et al.* (2020)
MUC5B+ club cells	Genetic association between MUC5B promoter polymorphism and PF patient survival.	Peljto *et al.* (2013)
MUC5B+ club cells	The rs35705950, 3 kb upstream of the MUC5B is found in 38% of PF cases but in only 9% of controls.	Seibold *et al.* (2011)
MUC5B+ club cells	Proportion of MUC5B+ club cells significantly is increased in PF patients.	Zuo *et al.* (2020)
SCGB3A2	Molecular phenotype of SCGB3A2 club cells was altered in PF lungs.	Zuo *et al.* (2020)
SCGB3A2	Experiment in Scgb3a2-transgenic mouse shows SCGB3A2 is an anti-fibrotic agent.	Cai and Kimura (2015)

#### 3.3.2 Results on MS

**Fig. 5.**
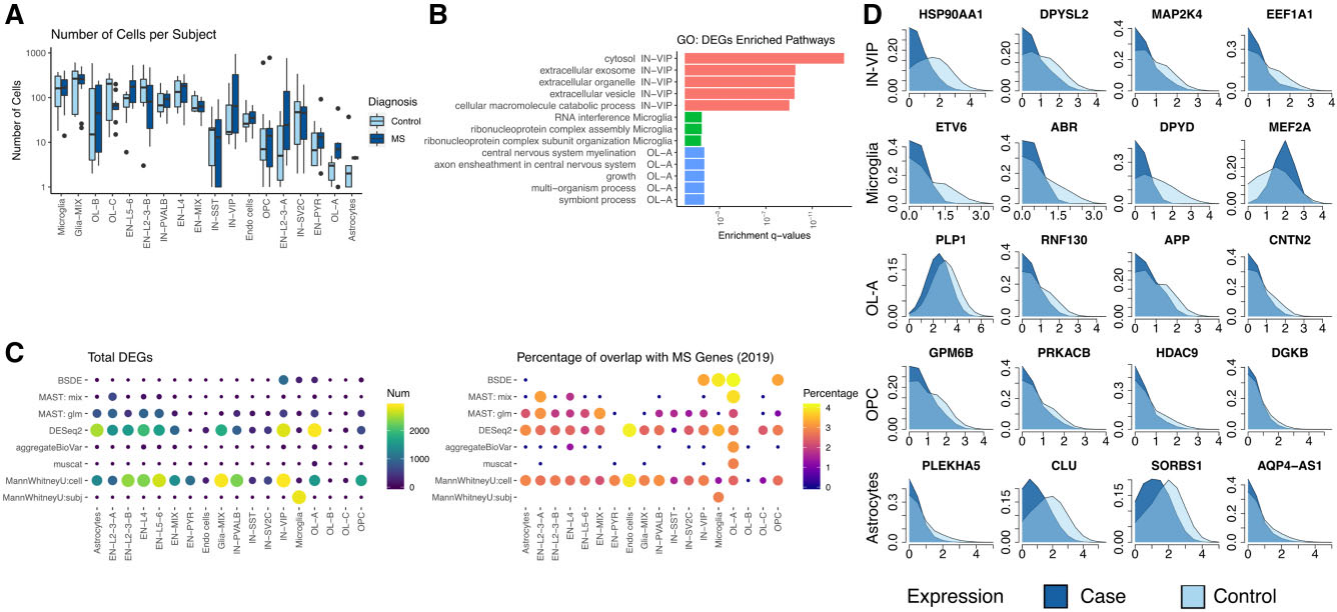
Analysis of the multiple sclerosis (MS) dataset. (**A**) Subject-level distributions of the number of cells from case and control samples. (**B**) Top cell types with the largest number of differentially expressed genes (DEGs) detected by BSDE. (**C**) Left: the total number of DEGs identified by each method. Right: the percentage of identified DEGs that overlap with the GWAS findings reported in Patsopoulos *et al.* (2019). (**D**) Case and control Wasserstein barycenter distributions for the top four differentially expressed genes (log-transformed expression)

The results are summarized in Figure 5. Figure 5A shows the subject-level distributions of the number of cells from case and control samples. No significant difference (*P*-value = 0.71, two-sided Bonferroni-corrected *t*-test) in the numbers of cells is observed between case and control. Figure 5B displays the top eight cell types with the largest DEGs. Signals are enriched in cell types IN-VIP, microglia, OL-A, OPC and astrocytes. Among these cell types, the significant pathways of GO enrichment analysis are reported in Figure 5C. Further, in Figure 5D, we contrast the case and the control Wasserstein barycenter distributions for the top four DEGs in five cell types.

##### Support from the literature

MS is one of the most common demyelinating diseases of the central nervous system. BSDE successfully detects cell-type-specific DEGs in L2-L3 EN, OL-A, IN-VIP, astrocytes, microglia and OPC, whose pathological roles have been established in the literature; see Table 2 and Supplementary Table S2 for more details. Additionally, we correlate the DEGs identified by BSDE and other methods with those reported by Patsopoulos *et al.* (2019) from a GWAS study. In that study, the International Multiple Sclerosis Genetics Consortium identified more than 233 MS risk loci from more than 47 000 cases and 68 000 controls; see Figure 5C. For the aforementioned cell types where BSDE finds the strongest signal, the findings seem to achieve a high percentage of overlap with the GWAS study.

**Table 2.** Previous reports of DEG-enriched cell types in MS identified by BSDE

Cell type	Previous reports	References
OL-A	Oligodendrocytes get the $H_{2}O_{2}$ produced from activated microglia through oxidative burst, and accumulate toxic Fe^2+^, which leads to the cell death and release of more Fe^2+^ to the environment.	Lassmann *et al.* (2012) and Nikić *et al.* (2011)
Astrocytes	The astrocytes are active for the lesion formation at the early stage of MS. They form a glial scar when the demyelination is completed at the later stage of MS.	Ponath *et al.* (2018)
OPC	MS is associated with the inhabitation of the differentiation of OPCs to mature oligodendrocytes, which are required for remyelination and disease remission.	Deshmukh *et al.* (2013) and Boyd *et al.* (2013)
Microglia	Microglial activation is associated with tissue injury in progressive MS.	Prineas *et al.* (2001)
IN-VIP	The experimental autoimmune encephalomyelitis (EAE) in mouse is an inflammatory autoimmune demyelinating disease of the central nervous system, which shares similarity to MS pathologically and clinically. Neuropeptide VIP protects against EAE by downregulating the inflammation and T-helper type-1 driven autoreactive response of MS.	Miller *et al.* (2010)

## 4 Discussion

Traditionally, scRNAseq datasets are collected from many cells of different cell types, but from only a few individuals, due to the high cost of sequencing and technical limitations. Consequently, most methods developed for scRNAseq analysis are focused on differential expression across cell types, instead of that between case and control individuals. However, as single-cell sequencing becomes cheaper and easier to use, an increasing number of datasets from case-control studies, especially those related to complex human diseases such as autism (Velmeshev *et al.*, 2019), PF (Habermann *et al.*, 2020) and MS (Schirmer *et al.*, 2019), are now available to us. Our method is developed to extend DE analysis to these emerging datasets, which could play a vital role in biomedical research.

A major challenge of such analysis is to compare individual-level distributions between case and control. Traditionally, parametric models (e.g. log-normal, Poisson, zero-inflated negative binomial) are developed to fit these distributions, through which the comparison can be performed with parametric two-sample tests. However, the control of type-I error is not guaranteed if the model is misspecified, or when a naive likelihood ratio test is applied to mixed-effect models (see Section 3.2). In fact, misspecification is highly likely for real datasets, where batch effects, heterogeneity and dropouts are frequently observed. Through a fully nonparametric approach based on permutation tests and optimal transport, our method is free from these issues. Further, subject-level distributions within case/control are aggregated via Wasserstein barycenter, a type of distributional averaging that takes account of the metric information in data, i.e., the difference in expression levels. Compared to other types of averaging (e.g. arithmetic) that ignore the metric information, the resulting aggregated distributions are much more informative; see Figures 4D and 5D. Additionally, BSDE is computationally affordable thanks to recent developments of fast algorithms for (entropy-regularized) optimal transport (Altschuler *et al.*, 2017; Cuturi, 2013; Cuturi and Doucet, 2014).

Data-driven methods for identifying differential expression provide important guidelines by suggesting candidate genes for further experimental studies. To this end, a short list of key genes is more valuable than a long list of irrelevant genes. With its nonparametric flexibility, strict type-I error guarantee and excellent detection power, BSDE is applicable to a wide range of DE analyses for case–control studies.

*Financial Support*: none declared.

*Conflict of Interest*: none declared.

## Data availability

The two single-cell sequencing datasets analyzed in the paper can be download with UCSC cell browser from cells.ucsc.edu/?ds=ms and cells.ucsc.edu/?ds=lung-pf-control.

## Supplementary Material

btac171_Supplementary_Data
