# Supplementary material for: BSDE: barycenter single-cell differential expression for case–control studies
Source: Bioinformatics. 2022 Mar 25;38(10):2765–72. doi: 10.1093/bioinformatics/btac171 (PMC9113363; doi:10.1093/bioinformatics/btac171)
Supplement: btac171_Supplementary_Data [file btac171_supplementary_data.pdf]

Supplementary Materials to "BSDE: Barycenter Single-Cell Differential Expression for Case-Control Studies"

Mengqi Zhang F. Richard Guo

1 Analysis of pulmonary fibrosis (PF) and multiple sclerosis (MS)

Tables S1 and S2 list additional support from the literature for differentially expressed genes identified by BSDE in PF and MS respectively. The summaries are taken from `genecards.org` (Safran et al., 2010).

Table S1: Previous reports of differentially expressed genes in PF identified by BSDE

Symbol	Description	GeneCards Summary	Association to PF	References
HLA-C	Major Histocompatibility Complex, Class I, C	It presents peptides derived from endoplasmic reticulum lumen in the immune system.	Genetic analysis shows that HLA-C group 1 homozygosity are associated with the presence of bronchiectasis. No record of association between HLA-C and PF by now.	Boyton et al. (2006)
LDHA	Lactate Dehydrogenase A	The protein catalyzes the conversion of L-lactate and NAD to pyruvate and NADH in the final step of anaerobic glycolysis.	Experiments on mice with genetic silencing of LDHA suggest that the inhibition of LDHA protects against radiation-induced PF.	Judge et al. (2015, 2017)
S100A10	S100 Calcium Binding Protein A10	This gene is involved in the regulation of a number of cellular processes such as cell cycle progression and differentiation. It is related to dissolution of fibrin clot and response to elevated platelet cytosolic Ca2+.	No report found about its association with PF.	

Continued on the next page

Table S1, continued

Symbol	Description	GeneCards Summary	Association to PF	References
FTL	Ferritin Light Chain	This gene encodes the light subunit of the ferritin protein, which stores iron in a soluble and nontoxic state. Disfunction of this light chain is associated with several neurodegenerative diseases. It has some multiple pseudogenes.	IPF patients exhibit iron aggregation in their lungs. The expression of FTL is also increased in their pulmonary fibroblasts. The accumulation of iron is also observed in the bleomycin (BLM)-induced PF mouse model.	Zhu et al. (2021)
PRDX1	Peroxiredoxin 1	Thiol-specific peroxidase that catalyzes the reduction of hydrogen peroxide and organic hydroperoxides to water and alcohols, respectively. Protect cell from oxidative stress by detoxifying peroxides and as sensor of hydrogen peroxide-mediated signaling events.	Mouse experiments suggest that PRDX1 regulates JNK kinase signaling to inhibit the cancer-associated fibroblasis-like phenotypes. PRDX1 might act as a protective role in bleomycin (BLM)-induced PF mice.	Jezierska-Drutel et al. (2019); Kikuchi et al. (2011)
RBM3	RNA Binding Motif Protein 3	This gene encode a mRNA binding protein that enhances global protein synthesis at both physiological and mild hypothermic temperatures. Expression of this gene is induced by cold shock and low oxygen tension.	Experiments in RBM3 gene knockout mice suggest that RBM3 downregulates innate lymphoid cells and lung inflammation.	Badrani et al. (2020)

Table S2: Previous reports of differentially expressed genes in MS identified by BSDE

Symbol	Description	GeneCards Summary	Association to MS	References
HSP90AA1	Heat Shock Protein 90 Alpha Family Class A Member 1	This is a molecular chaperone helping with folding and quality control for kinds of proteins. It is related to apoptosis modulation and signaling and semaphorin interactions.	HSP90AA1 promotes autophagy via AKT-MTOR pathway. It is reported that drugs targeting the PI3K/Akt/mTOR pathway may be useful to impede disease progression and to promote the remyelination process, so to reverse disability in MS patients. The RNA inference HSP90AA1-AKT-MTOR knock down inhibits autophagy during the infection.	Mammana et al. (2018); Schirmer et al. (2019)
DPYSL2	Dihydropyrimidinase Like 2 or Collapsin Response Mediator Protein-2 (CRMP2).	This gene's product is involved in neuronal development and polarity, axon growth and guidance, neuronal growth cone collapse and cell migration. Alzheimer and neuronal ceroid lipofuscinosis are reported associated with this gene.	According to the transgenesis mouse model, the phosphorylation of CRMP-2 involves in axonal degeneration of multiple sclerosis.	Petratos et al. (2012)
ABR	ABR Activator of RhoGEF and GTPase	This gene encodes a protein containing a GTPase-activating protein domain, a domain found in members of the Rho family of GTP-binding proteins.	No report found about its association with MS.	
ETV6	ETS Variant Transcription Factor 6	ETV6 is involved in leukemia and congenital fibrosarcoma associated chromosomal rearrangements.	This gene is shown to be differentially expressed in MS compared to control on mice models.	Tseveleki et al. (2010)

Continued on the next page

Table S2, continued

Symbol	Description	GeneCards Summary	Association to MS	References
PLP1	Proteolipid Protein 1	This is the major myelin protein for the formation or maintenance of the multilamellar structure of myelin in the central nervous system.	Several genetic studies, including family analysis and association studies, link PLP1 mutations to MS in human.	Cloake et al. (2018); Warshawsky et al. (2005)
RNF130	Ring Finger Protein 130	It is related to innate immune system and may work during the programmed cell death of hematopoietic cells.	miR-340 is located in the intron of RNF130 and share the same promoter hypermethylation with RNF130. Their expressions are correlated. miR-340 is over-expressed in patients with MS. Based on the mouse experiments, MiR-340-5p inhibits gene PDCD4 to protect neurons against OGDR injury and influences PI3K/Akt signaling, which is related to MS.	Huang et al. (2021); Zheng et al. (2020); Schirmer et al. (2019)
GPM6B	Glycoprotein M6B	This gene encodes a membrane glycoprotein that belongs to the proteolipid protein family which are expressed in most brain regions for cellular housekeeping functions. This protein may be related to osteoblast differentiation.	By targeting the GPM6B gene in crossbreeding experiments, single-mutant mice lacking either PLP or M6B are fully myelinated, while double mutants remain severely hypomyelinated with enhanced neurodegeneration and premature death.	Werner et al. (2013)

Continued on the next page

Table S2, continued

Symbol	Description	GeneCards Summary	Association to MS	References
PRKACB	Protein Kinase CAMP-Activated Catalytic Subunit Beta	The protein encoded by this gene is a catalytic subunit of cAMP (cyclic AMP)-dependent protein kinase, which mediates signaling though cAMP. cAMP signaling is important to a number of processes, including cell proliferation and differentiation.	No report found about its association with MS.	
PLEKHA5	Pleckstrin Homology Domain containing A5	This gene encodes a protein associated with Cleft Lip. It is related to metabolic pathways.	This gene shows up as dysregulated circulating nucleic acids from patients with relapsing MS compared to healthy controls. There are rare reports about the expression of this gene within the brain.	Beck et al. (2010)
CLU	Clusterin	This gene encodes a secreted chaperone under some stress conditions as well as in the cell cytosol. It has been suggested to be involved in cell death, tumor progression, and neurodegenerative disorders.	In situ expressions show greater expression of CLU in white matter lesions than in normal-appearing regions in MS patients and in the marmosets, primarily in or adjacent to perivascular spaces and inflammatory infiltrates.	van Luijn et al. (2016); Chen et al. (2021)

2 Additional simulation results

In the following pages, we include additional simulation results from different settings, where the number of individuals is varied from $\{10, 20, 40\}$ and the number of cells is varied from $\{20, 50, 100, 200, 300, 400\}$. One can see that, across all the settings, BSDE achieves a reasonable detection power while maintaining the type-I error at the prescribed level 0.05.

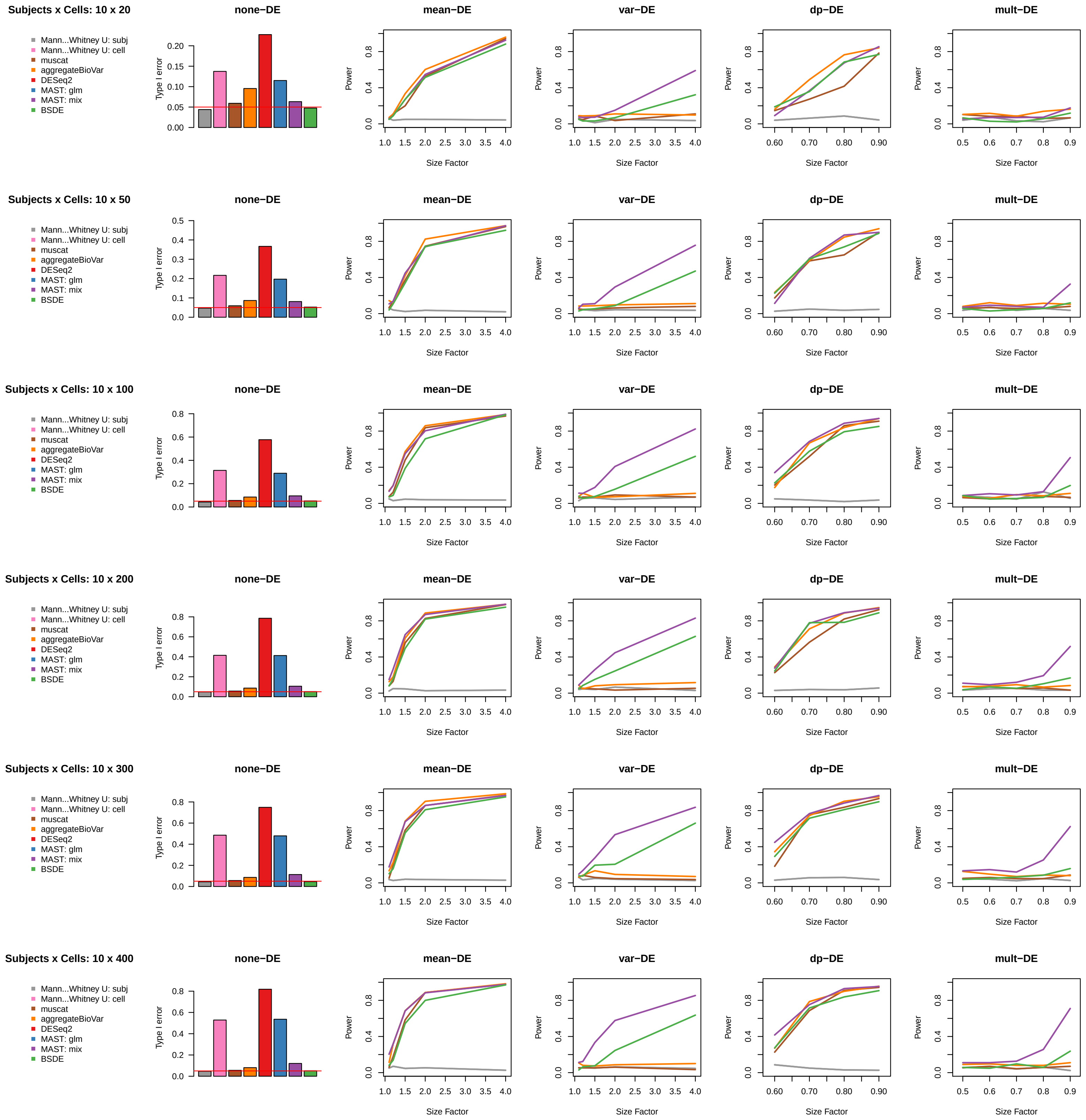
Subjects x Cells: 10 x 20
Subjects x Cells: 10 x 50
Subjects x Cells: 10 x 100
Subjects x Cells: 10 x 200
Subjects x Cells: 10 x 300
Subjects x Cells: 10 x 400
none−DE
mean−DE
var−DE
dp−DE
mult−DE
Mann...Whitney U: subj
Mann...Whitney U: cell
muscat
aggregateBioVar
DESeq2
MAST: glm
MAST: mix
BSDE
Type I error
Power
Size Factor

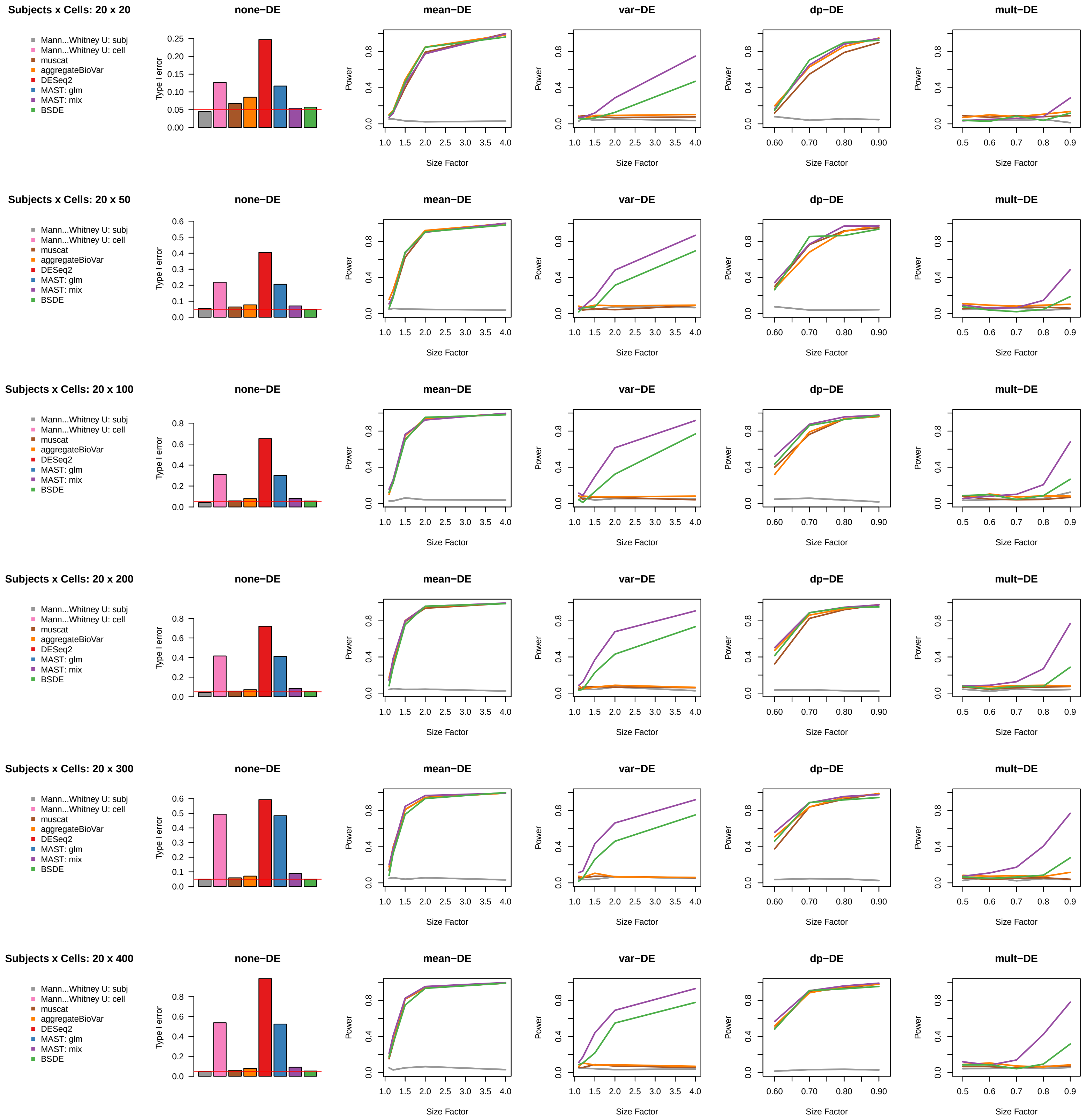

Subjects x Cells: 20 x 20
Subjects x Cells: 20 x 50
Subjects x Cells: 20 x 100
Subjects x Cells: 20 x 200
Subjects x Cells: 20 x 300
Subjects x Cells: 20 x 400
none−DE
mean−DE
var−DE
dp−DE
mult−DE
Mann...Whitney U: subj
Mann...Whitney U: cell
muscat
aggregateBioVar
DESeq2
MAST: glm
MAST: mix
BSDE
Type I error
Power
Size Factor

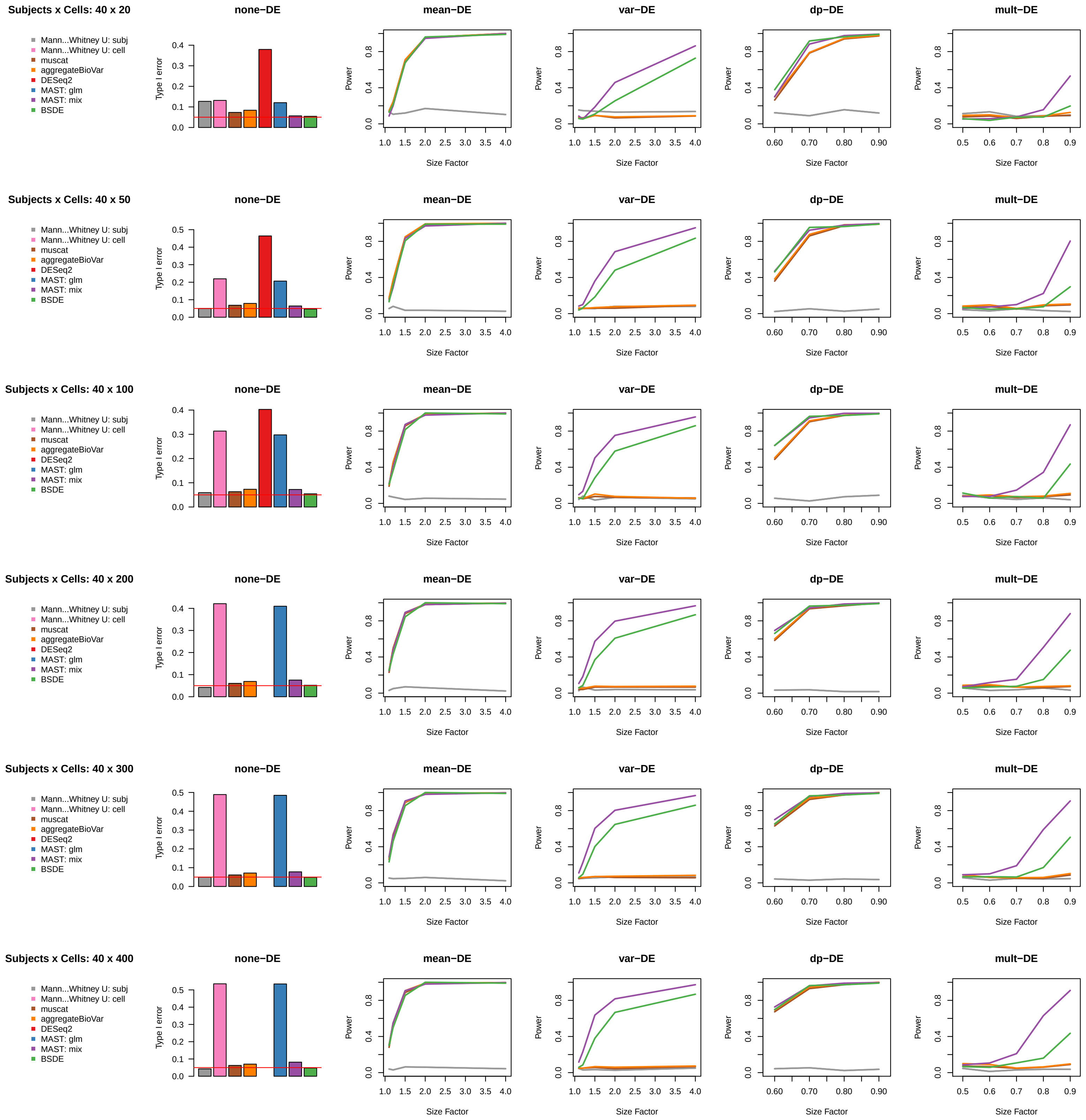
Subjects x Cells: 40 x 20
Subjects x Cells: 40 x 50
Subjects x Cells: 40 x 100
Subjects x Cells: 40 x 200
Subjects x Cells: 40 x 300
Subjects x Cells: 40 x 400
Mann...Whitney U: subj
Mann...Whitney U: cell
muscat
aggregateBioVar
DESeq2
MAST: glm
MAST: mix
BSDE
none−DE
mean−DE
var−DE
dp−DE
mult−DE
Type I error
Power
Size Factor
0.0
0.1
0.2
0.3
0.4
0.5
0.8
1.0 1.5 2.0 2.5 3.0 3.5 4.0
0.60 0.70 0.80 0.90
0.5 0.6 0.7 0.8 0.9

References

Jana Badrani, Michael Amadeo, Kellen Cavagnero, Luay Naji, Sean Lund, Rachel Baum, Naseem Khorram, Julie Pilotte, Peter Vanderklish, and Taylor Doherty. The RNA-Binding protein RBM3 negatively regulates group 2 innate lymphoid cells (ILC2s) and lung eosinophilia. *Journal of Allergy and Clinical Immunology*, 145(2):AB1, 2020.

Julia Beck, Howard B Urnovitz, Marina Saresella, Domenico Caputo, Mario Clerici, William M Mitchell, and Ekkehard Schütz. Serum DNA motifs predict disease and clinical status in multiple sclerosis. *The Journal of Molecular Diagnostics*, 12(3):312–319, 2010.

Rosemary J Boyton, John Smith, Rosemary Ward, Meinir Jones, Lorraine Ozerovitch, Robert Wilson, Marlene Rose, John Trowsdale, and Daniel M Altmann. HLA-C and killer cell immunoglobulin-like receptor genes in idiopathic bronchiectasis. *American Journal of Respiratory and Critical Care Medicine*, 173(3):327–333, 2006.

Fading Chen, Dan B Swartzlander, Anamitra Ghosh, John D Fryer, Baiping Wang, and Hui Zheng. Clusterin secreted from astrocyte promotes excitatory synaptic transmission and ameliorates Alzheimer's disease neuropathology. *Molecular Neurodegeneration*, 16(1):1–16, 2021.

Nancy C Cloake, Jun Yan, Atefeh Aminian, Michael P Pender, and Judith M Greer. PLP1 mutations in patients with multiple sclerosis: identification of a new mutation and potential pathogenicity of the mutations. *Journal of Clinical Medicine*, 7(10):342, 2018.

Zheng Huang, Yesha Xu, Maoping Wan, Xixi Zeng, and Jianmin Wu. mir-340: A multifunctional role in human malignant diseases. *International Journal of Biological Sciences*, 17(1):236, 2021.

Agnieszka Jezierska-Drutel, Shireen Attaran, Barbara L Hopkins, John J Skoko, Steven A Rosenzweig, and Carola A Neumann. The peroxidase PRDX1 inhibits the activated phenotype in mammary fibroblasts through regulating c-Jun N-terminal kinases. *BMC Cancer*, 19(1):1–13, 2019.

Jennifer L Judge, Kristina M Owens, Stephen J Pollock, Collynn F Woeller, Thomas H Thatcher, Jacqueline P Williams, Richard P Phipps, Patricia J Sime, and Robert Matthew Kottmann. Ionizing radiation induces myofibroblast differentiation via lactate dehydrogenase. *American Journal of Physiology-Lung Cellular and Molecular Physiology*, 309(8):L879–L887, 2015.

Jennifer L Judge, Shannon H Lacy, Wei-Yao Ku, Kristina M Owens, Eric Hernady, Thomas H Thatcher, Jacqueline P Williams, Richard P Phipps, Patricia J Sime, and R Matthew Kottmann. The lactate dehydrogenase inhibitor gossypol inhibits radiation-induced pulmonary fibrosis. *Radiation research*, 188(1):35–43, 2017.

Norihiro Kikuchi, Yukio Ishii, Yuko Morishima, Yuichi Yageta, Norihiro Haraguchi, Tadahiro Yamadori, Hironori Masuko, Tohru Sakamoto, Toru Yanagawa, and Eiji Warabi. Aggravation of bleomycin-induced pulmonary inflammation and fibrosis in mice lacking peroxiredoxin I. *American Journal of Respiratory Cell and Molecular Biology*, 45(3):600–609, 2011.

Santa Mammana, Placido Bramanti, Emanuela Mazzon, Eugenio Cavalli, Maria Sofia Basile, Paolo Fagone, Maria Cristina Petralia, James Andrew McCubrey, Ferdinando Nicoletti, and Katia

Mangano. Preclinical evaluation of the PI3K/Akt/mTOR pathway in animal models of multiple sclerosis. *Oncotarget*, 9(9):8263, 2018.

Steven Petratos, Ezgi Ozturk, Michael F Azari, Rachel Kenny, Jae Young Lee, Kylie A Magee, Alan R Harvey, Courtney McDonald, Kasra Taghian, and Leon Moussa. Limiting multiple sclerosis related axonopathy by blocking Nogo receptor and CRMP-2 phosphorylation. *Brain*, 135(6):1794–1818, 2012.

Marilyn Safran, Irina Dalah, Justin Alexander, Naomi Rosen, Tsippi Iny Stein, Michael Shmoish, Noam Nativ, Iris Bahir, Tirza Doniger, and Hagit Krug. Genecards Version 3: the human gene integrator. *Database*, 2010, 2010.

Lucas Schirmer, Dmitry Velmeshev, Staffan Holmqvist, Max Kaufmann, Sebastian Werneburg, Diane Jung, Stephanie Vistnes, John H Stockley, Adam Young, and Maike Steindel. Neuronal vulnerability and multilineage diversity in multiple sclerosis. *Nature*, 573(7772):75–82, 2019.

Vivian Tseveleki, Renee Rubio, Sotiris-Spyros Vamvakas, Joseph White, Era Taoufik, Edwige Petit, John Quackenbush, and Lesley Probert. Comparative gene expression analysis in mouse models for multiple sclerosis, Alzheimer's disease and stroke for identifying commonly regulated and disease-specific gene changes. *Genomics*, 96(2):82–91, 2010.

Marvin M van Luijn, Marjan van Meurs, Marcel P Stoop, Evert Verbraak, Annet F Wierenga-Wolf, Marie-José Melief, Karim L Kreft, Robert M Verdijk, Bert A 't Hart, and Theo M Luider. Elevated expression of the cerebrospinal fluid disease markers chromogranin A and clusterin in astrocytes of multiple sclerosis white matter lesions. *Journal of Neuropathology & Experimental Neurology*, 75(1):86–98, 2016.

Ilka Warshawsky, Richard A Rudick, Susan M Staugaitis, and Marvin R Natowicz. Primary progressive multiple sclerosis as a phenotype of a PLP1 gene mutation. *Annals of Neurology*, 58(3): 470–473, 2005.

Hauke B Werner, Eva-Maria Krämer-Albers, Nicola Strenzke, Gesine Saher, Stefan Tenzer, Yoshiko Ohno-Iwashita, Patricia De Monasterio-Schrader, Wiebke Möbius, Tobias Moser, and Ian R Griffiths. A critical role for the cholesterol-associated proteolipids PLP and M6B in myelination of the central nervous system. *Glia*, 61(4):567–586, 2013.

Yake Zheng, Peng Zhao, Yajun Lian, Shuang Li, Yuan Chen, and Lihao Li. MiR-340-5p alleviates oxygen-glucose deprivation/reoxygenation-induced neuronal injury via PI3K/Akt activation by targeting PDCD4. *Neurochemistry International*, 134:104650, 2020.

Yumeng Zhu, Jing Chang, Ke Tan, Steven K Huang, Xin Liu, Xiaofan Wang, Mengshu Cao, Hongmin Zhang, Shuxin Li, and Xianglin Duan. Clioquinol attenuates pulmonary fibrosis through inactivation of fibroblasts via iron chelation. *American Journal of Respiratory Cell and Molecular Biology*, 2021.
